# Coronary embolism due to probable clinical bioprosthetic aortic valve thrombosis: a case report

**DOI:** 10.1186/s12872-023-03359-9

**Published:** 2023-06-26

**Authors:** Roshan A Ananda, Zhihua Zhang

**Affiliations:** 1 Department of Cardiology, Mackay Base Hospital, Queensland, Australia; 2 James Cook University, Queensland, Australia

**Keywords:** Coronary embolism, Bioprosthetic aortic valve, Surgical aortic valve replacement, Bioprosthetic valve thrombosis, Anticoagulation, Case report

## Abstract

**Background:**

Coronary embolism is a relatively rare but important non-atherosclerotic cause of acute coronary syndrome, mainly caused by atrial fibrillation and mechanical heart valve thrombosis due to subtherapeutic anticoagulation. There have been increasing reports of bioprosthetic valve thrombosis (BPVT), but thromboembolic events are rare and mainly affect the cerebrovascular system. Coronary embolism is an extremely rare complication of BPVT.

**Case presentation:**

A 64-year-old male presented with non-ST-Elevation myocardial infarction (NSTEMI) to an Australian regional health service. Three years ago, he had undergone Bentall procedure with bioprosthetic aortic valve replacement for severe aortic regurgitation and significant aortic root dilatation. Diagnostic coronary angiography revealed embolic occlusion of first diagonal branch in the absence of underlying atherosclerosis. Prior to NSTEMI presentation, the patient was clinically asymptomatic apart from the progressive increase in transaortic mean pressure gradient on transthoracic echocardiography which was first detected seven months after surgical aortic valve replacement. Transoesophageal echocardiography showed restrictions of the aortic leaflet opening but no evidence of mass or vegetation. After eight weeks of warfarin therapy, the raised aortic valve gradient returned to normal. Lifelong warfarin was prescribed, and patient remained clinically well at 39-month follow-up.

**Conclusion:**

We experienced a case of coronary embolism in a patient with probable BPVT. Reversible bioprosthetic valve hemodynamic deterioration after anticoagulation strongly supports the diagnosis in the absence of histopathology. Early moderate-to-severe hemodynamic valve deterioration warrants further investigations, including cardiac computed tomography and sequential echocardiography, to investigate for probable BPVT and consideration of timely anticoagulation initiation to prevent thromboembolic events.

**Supplementary Information:**

The online version contains supplementary material available at 10.1186/s12872-023-03359-9.

## Background

Coronary embolism (CE) is a relatively rare but important non-atherosclerotic cause of acute coronary syndrome, accounting for 2.9% of de novo acute myocardial infarction [1]. It is mostly caused by atrial fibrillation (73%) but can be secondary to cardiomyopathy, valvular heart disease, malignancy, or infective endocarditis [1]. The management of CE can be challenging due to its multifactorial aetiologies, and often requires individualized therapeutic approach [1].

Bioprosthetic heart valves are known to be less thrombogenic, but there have been increasing reports of their association with subclinical bioprosthetic valve thrombosis (BPVT) and are more common in transcatheter than surgical replacement [2, 3]. Subclinical BPVT is described as a thin layer of thrombus in the cusp of one or more bioprosthetic valves which restricts the leaflet motion [3]. Although the risk of SBPVT is relatively low, it is associated with increased risk of cerebral thromboembolic events if left untreated [2, 4]. Traditionally described as the presence of thrombus on echocardiography or imaging, clinical BPVT has been redefined as the presence of clinical sequelae of a thromboembolic event or worsening valve stenosis/ regurgitation and either imaging evidence of thrombus or moderate-to-severe hemodynamic valve deterioration [3, 5]. We report a case of non-ST-elevation myocardial infarction (NSTEMI) secondary to coronary embolism in a patient with probable clinical bioprosthetic aortic valve thrombosis.

## Case presentation

**Fig. 1:**
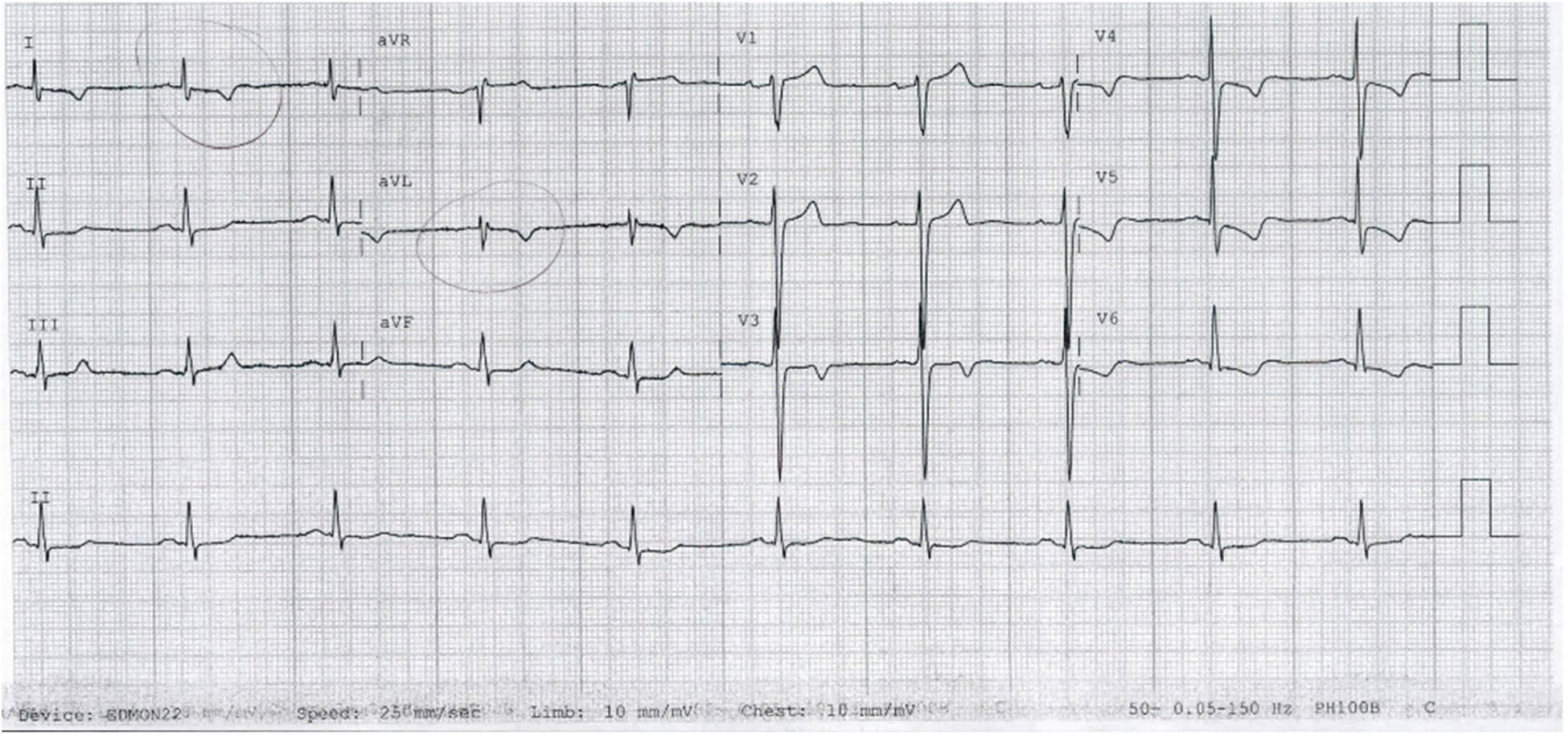
Electrocardiogram at presentation

A 64-year-old male presented with chest pain to a regional health service. He was afebrile and vital signs showed blood pressure of 144/91 mmHg and heart rate of 61 beats per minute. Cardiac examination revealed non-radiating grade 3 ejection systolic murmur in the aortic region and no clinical signs of cardiac failure. He had undergone surgical bioprosthetic aortic valve replacement for severe aortic regurgitation three years ago. His cardiovascular risk factors include hypertension, past smoking history, valvular heart disease and history of intracerebral haemorrhage. He has no previous history of cardiac arrhythmias, malignancy, coagulation disorders or intravenous drug use. The initial electrocardiogram showed sinus rhythm with lateral ST-depression and T-wave inversions (Fig. 1). There were no dynamic changes in the subsequent electrocardiograms. Initial laboratory tests showed haemoglobin of 178 g/L, white cell count of 7.6 × 10^9^/L, neutrophil of 5.4 × 10^9^/L, platelet of 163,000/μL, creatinine of 83 μmol/L, high-sensitivity troponin of 208 ng/L, and two sets of negative blood cultures. Oral aspirin 300 mg, ticagrelor 180 mg and subcutaneous enoxaparin 100 mg injection were administered. He was admitted to cardiac care unit with intermediate-risk NSTEMI (GRACE score = 105) and dual antiplatelet therapy (DAPT). Ticagrelor was switched to clopidogrel due to reduced risk of intracerebral haemorrhage. Inpatient cardiac telemetry showed sinus rhythm with infrequent isolated supraventricular and ventricular ectopic over 72 h. There was also no evidence of paroxysmal atrial fibrillation on 24-h Holter monitor.

**Fig. 2:**
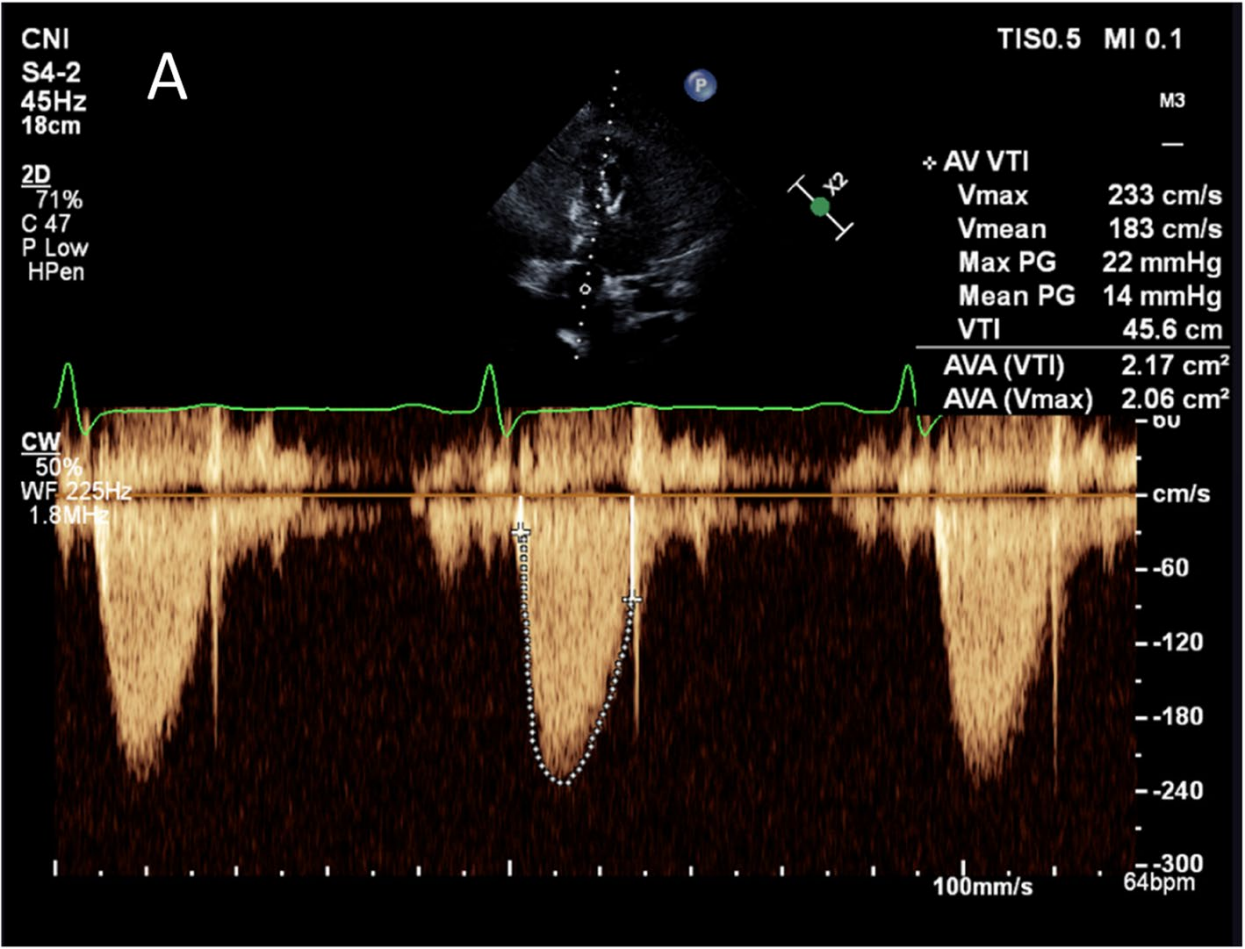
Postoperative transthoracic echocardiography. Normal aortic bioprosthetic valve hemodynamic

**Fig. 3:**
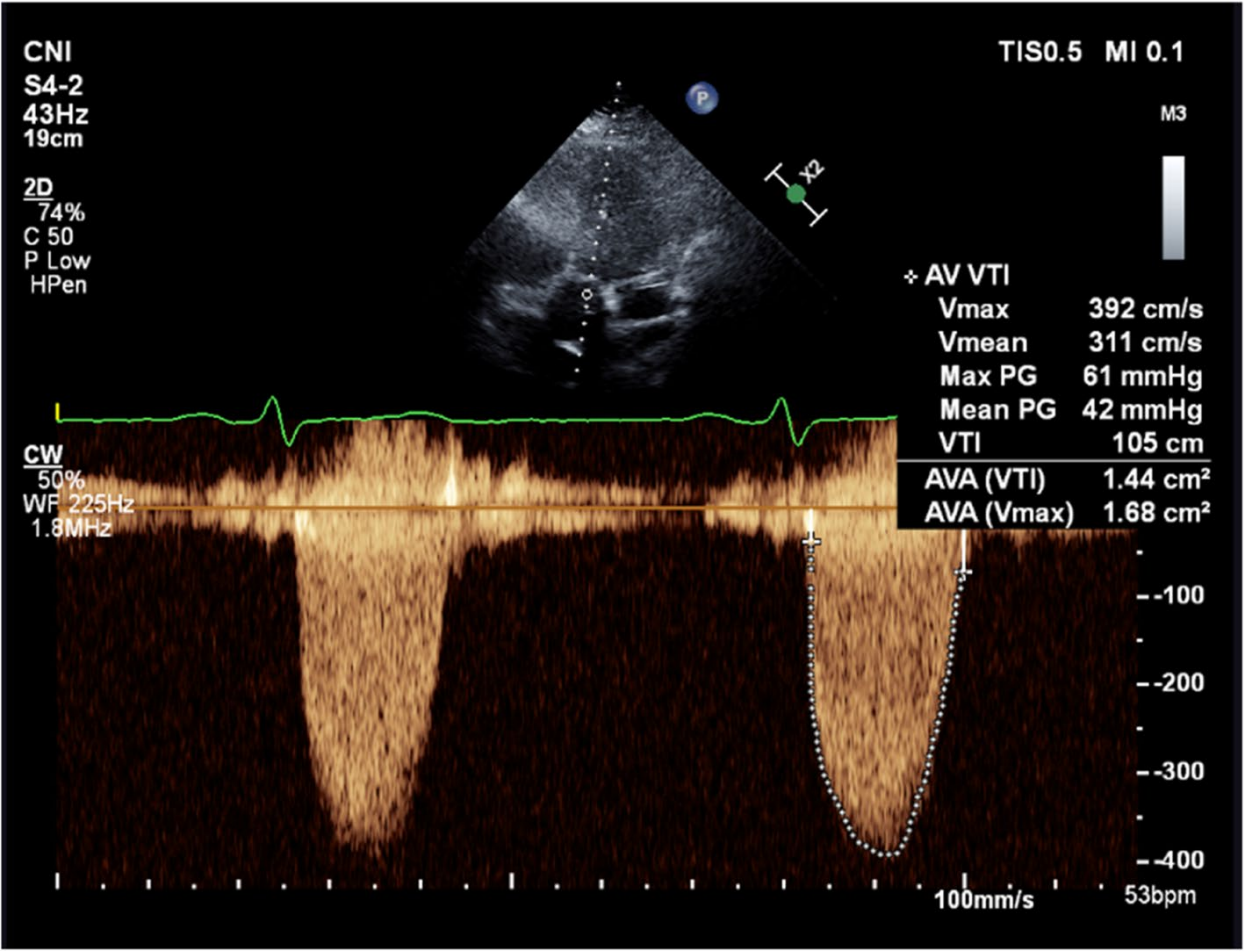
Transthoracic echocardiography prior to clinical presentation. Elevated aortic valve hemodynamic on transthoracic echocardiography 2.5 years following surgery

Three years ago, he had undergone Bentall procedure (32 mm Valsalva conduit) with bioprosthetic aortic valve replacement (Epic™ 27 mm aortic valve) for bicuspid aortic valve with severe aortic regurgitation and significant aortic root dilatation. After the uneventful surgery, he was started on regular aspirin 100 mg. Postoperative transthoracic echocardiography (TTE) showed normal aortic valve hemodynamic with no evidence of prosthesis-patient mismatch (Fig. 2; Table 1). Raised transaortic mean pressure gradient (22 mmHg) was first detected seven months after bioprosthesis implantation. While he remained asymptomatic despite having a physically demanding job as a builder for years, annual TTE surveillance demonstrated gradual increase in aortic valve hemodynamics (EF 55–65%; V_max_ 2.92–3.92 m/s; PPG 34-61 mmHg; MPG 22-42 mmHg; Fig. 3; Table 1). Although there was no mass or vegetation visualized on transoesophageal echocardiography, restrictions of the aortic leaflet opening and non-specific leaflet thickening contributed to the raised transaortic gradient (Additional file 1). There was no evidence of prosthesis-patient mismatch, paravalvular regurgitation, interatrial or interventricular shunt on TOE and annual TTE with colour flow doppler.

**Table 1:** The trend of bioprosthetic valve hemodynamic based on echocardiography

	Feb 2017	Aug 2017	Sep 2018	Apr 2019	Jun 2019	Aug 2019	Nov 2019 (Event)	Jan 2020	Jul 2020	Jul 2021	JAN 2022	Jan 2023
**V**_**max**_ **(m/s)**	2.33	2.92	3.30	3.92	4.40	3.90	3.17	2.41	2.30	2.54	2.20	2.32
**MPG (mmHg)**	14	22	24	42	51	37	22	13	10	12	11	12
**PPG (mmHg)**	22	34	43	61	78	61	40	23	21	26	21	21
**AVA (cm**^**2**^**)**	2.17	2.15	2.00	1.64	-	1.57	2.0	3.10	2.93	2.33	4.09	3.22
**DPI**	0.41	0.36	0.30	0.26	-	-	0.30	0.62	0.44	0.41	0.54	0.49

*AVA* Aortic valve area, *DPI* Dimensionless performance index, *MPG *Mean pressure gradient, *PPG *Peak pressure gradient, *V*_*max*_ Peak velocity

**Fig. 4:**
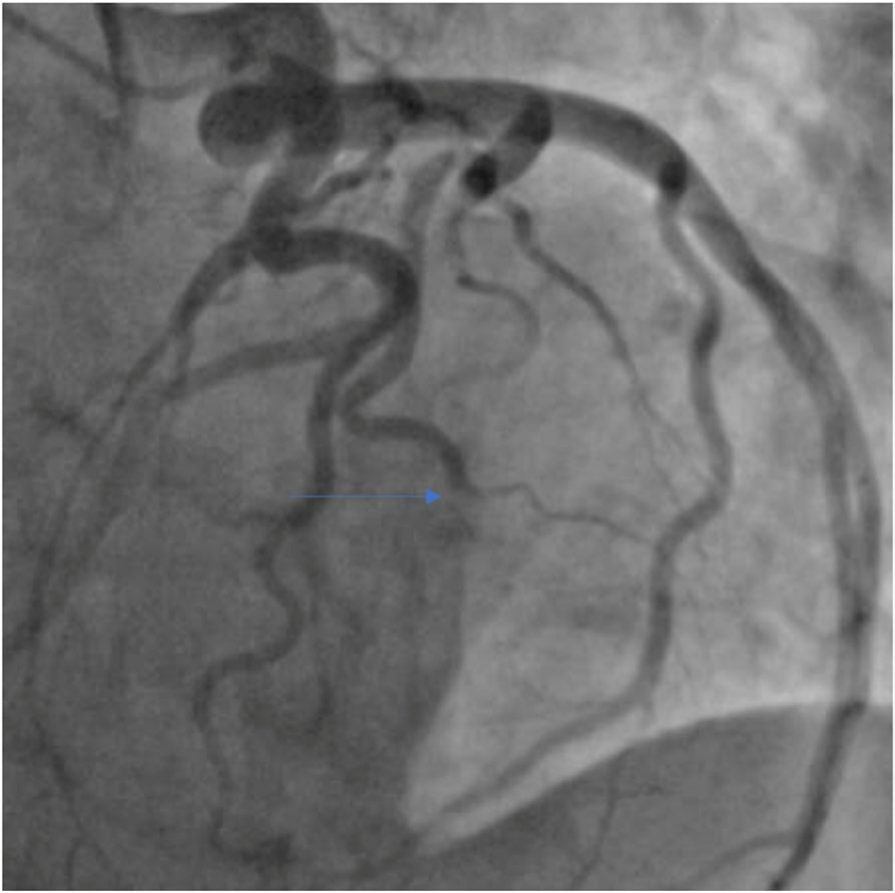
Coronary angiography at presentation. Left anterior oblique (LAO) cranial view of the coronary arteries. Blue arrow shows the embolus in the first diagonal branch

**Fig. 5:**
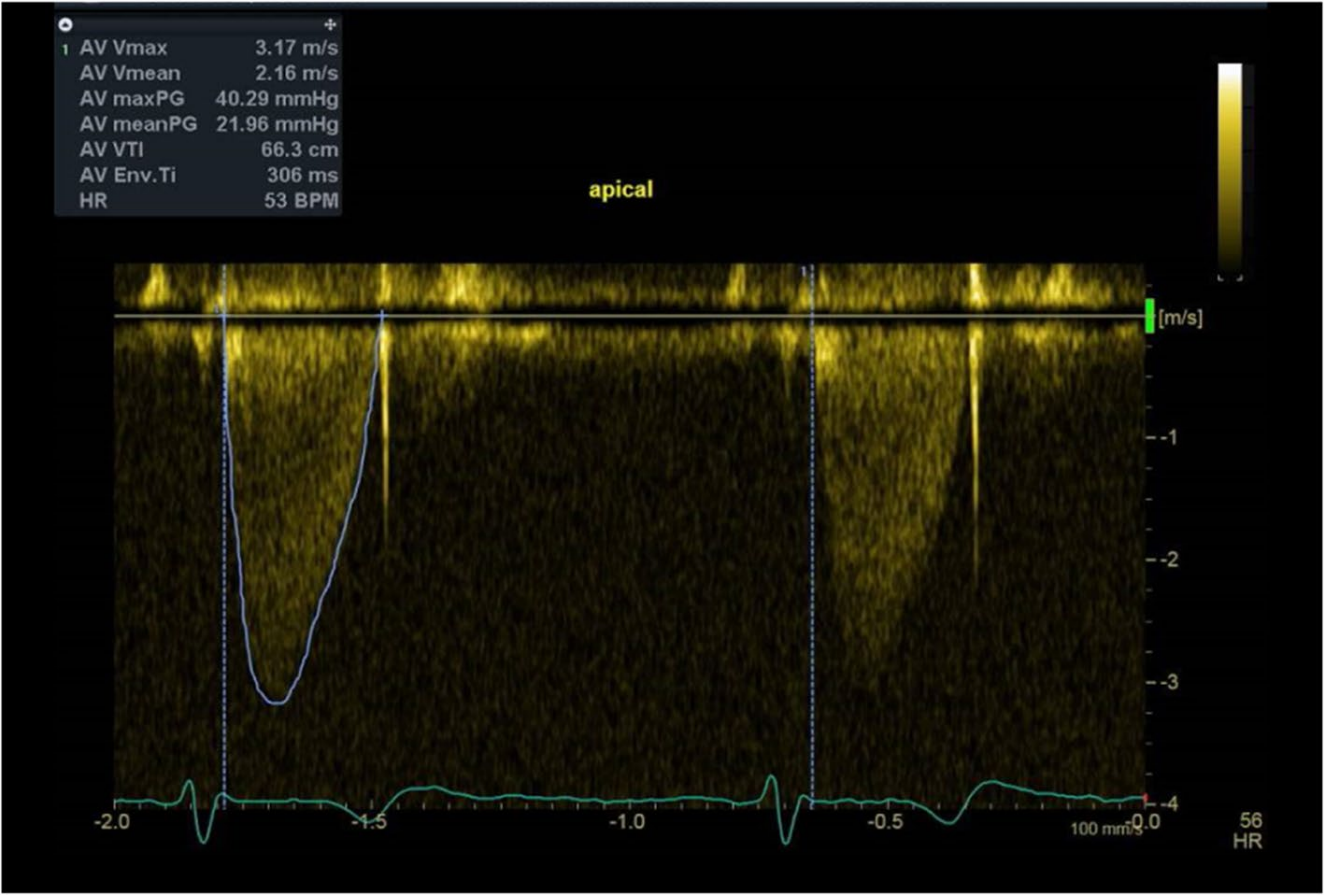
Transthoracic echocardiography at presentation. Raised transaortic gradient is likely underestimated by low quality Doppler signal of transthoracic echocardiography

Progressive increase in transaortic mean pressure gradient and NSTEMI presentation may indicate degenerative bioprosthetic valve, coronary embolism, or bioprosthetic valve thrombosis. Diagnostic coronary angiography revealed embolic occlusion of the first diagonal branch with no underlying atherosclerosis of the left main and left anterior descending coronary arteries (Fig. 4; Additional file 2). While a large aneurysm of the left main coronary artery was visualized, the right coronary artery appeared normal on aortogram. Raised transaortic gradient (PPG 40 mmHg; MPG 22 mmHg; Vmax 3.2 m/s; EF 45–50%; Fig. 5; Additional file 3) was likely underestimated by low quality Doppler signal of TTE on acute presentation. In view of cerebral aneurysm history and non-diagnostic computed tomography, magnetic resonance imaging of the brain was performed and revealed a small-sized cavernoma at the left basal ganglia.

**Fig. 6:**
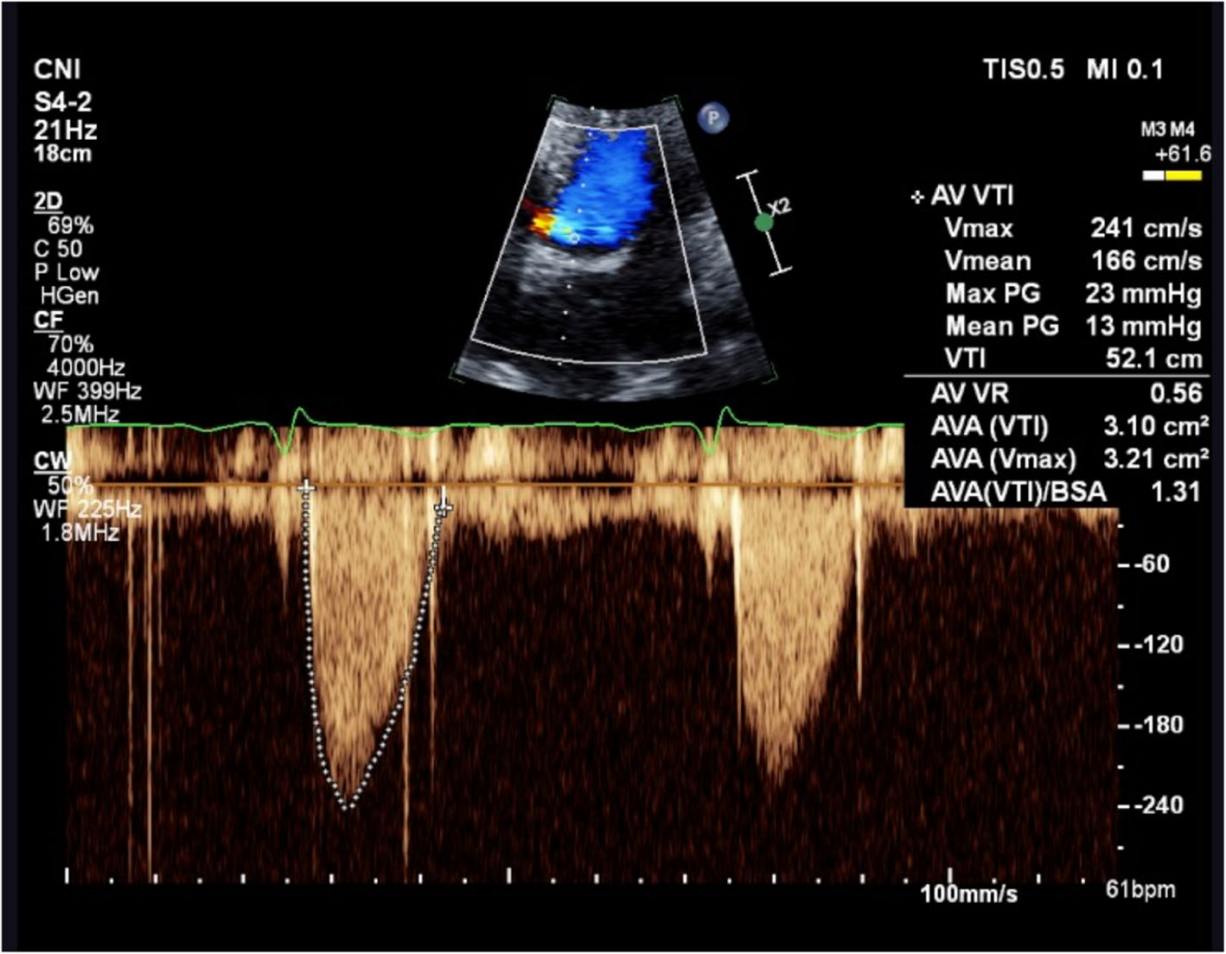
Transthoracic echocardiography after eight weeks of anticoagulation. Raised transaortic gradient returned to normal

Angiographic finding of coronary embolism raised the possibility of dislodged microthrombus from the abnormal tissue aortic valve. After extensive family discussion with neurosurgery and neurology opinions, DAPT was ceased, but warfarin was commenced with enoxaparin bridging and a target INR 2 to 3. The transaortic MPG improved to 13 mmHg after eight weeks of anticoagulation (Fig. 6). The patient remained clinically well with normal aortic valve hemodynamics at 39-month follow-up. Lifelong warfarin was prescribed.

## Discussion

The diagnosis and management of coronary embolism (CE) are challenging due to its multifactorial causative factors, and often require individualized therapeutic approach [1]. Urgent inpatient coronary angiography is essential to establish the diagnosis. Currently, there is no consensus on the management of CE. Routine thrombus aspiration of the infarct-related artery is not recommended due to the small increased risk of stroke with no mortality benefit [6]. Although high thrombus burden is an important risk factor for mortality, aspiration thrombectomy should only be considered if it alters percutaneous coronary intervention outcomes [6]. Small distal emboli should be managed with anticoagulation only [7]. CE is classified into direct, paradoxical, or iatrogenic [7]. Previous reports of valvular replacement-related CE were associated with echocardiographic finding of mobile thrombus suggestive of clinical BPVT, and most cases were due to subtherapeutic warfarin therapy for mechanical valves [1, 8]. Dislodge of microthrombus from the bioprosthetic aortic valve was the likely contributory factor to direct CE in our patient [7].

Probable diagnosis of clinical BPVT was formulated based on echocardiographic finding of severe transaortic hemodynamic deterioration and angiographic finding of CE, which represents clinical sequelae of a thromboembolic event [5]. Our patient achieved good response following eight weeks of warfarin with resolution of transaortic hemodynamic changes on follow-up TTE. Bioprosthetic heart valves are known to be less thrombogenic and long-term anticoagulation is often not required, but there have been increasing reports of subclinical BPVT in recent years [9]. To date, there is no consensus on the therapeutic approach for subclinical BPVT and the evidence is largely derived from non-randomised trials [2]. Subclinical BPVT is associated with increased risk of cerebral thromboembolic events if left untreated, but on the other hand, its natural history suggests that it may regress or progress spontaneously [4, 10]. Our patient received standard aspirin following bioprosthetic aortic valve replacement. As he remained asymptomatic despite a physically demanding job, watchful-waiting approach and annual TTE surveillance were carried out to monitor bioprosthetic valve hemodynamics. TOE was performed to evaluate the aetiology of progressive transaortic gradient deterioration in our patient. There was evidence of restricted aortic leaflet opening and non-specific leaflet thickening on TOE, but no features suggestive of vegetation, calcifications, or echogenous mass. Early degeneration of bioprosthetic valve was initially considered in our patient, but unfortunately, our case progressed into clinically significant valve thrombosis and suffered from a coronary thromboembolic event after two years of close surveillance.

It is clinically very challenging to differentiate subclinical BPVT from degenerative bioprosthetic valve based on the echocardiographic finding of deteriorating transaortic hemodynamic and the mainstay treatment of these conditions is very distinct. Both TOE and cardiac computed tomography (CT) provide good visualisation of leaflet motion and could differentiate thrombus and calcifications from degenerative valve by the differences in the attenuation of heart valve lesions [10]. However, in cases with non-visible thrombus, cardiac CT could provide more information on the leaflet morphology and raised the suspicion for microthrombus if there is evidence of hypo-attenuated leaflet thickening [2]. While BPVT can be managed with anticoagulation only, patients with degenerative bioprosthetic valve will require redo valve surgery or transcatheter valve-in-valve implantation [9]. Current guidelines recommend the use of Vitamin K antagonist in clinical or subclinical BPVT, but the treatment duration remains debatable and the long-term prognosis remains unknown [9, 11].

On presentation with NSTEMI, cardiac CT was not performed in our patient as CE diagnosis was confirmed by the evidence of hypoattenuating lesion on coronary angiography and severe hemodynamic valve deterioration on sequential echocardiography. Retrospectively, our patient was asymptomatic and had a six-month lead time between severe hemodynamic valve deterioration and coronary thromboembolic event. Cardiac CT would have provided additional cross-sectional assessment of the aortic valve morphology and prompted the consideration of anticoagulation initiation if suspicious for BPVT [2]. Despite the uncertain natural history and valve-related consequences of subclinical BPVT, trial of anticoagulation with follow-up echocardiography to assess for potential temporal resolution could potentially benefit patient with moderate-to-severe hemodynamic valve deterioration and probable clinical BPVT who remained hemodynamically stable to prevent thromboembolic event [12].

We present the first documented case of coronary embolism secondary to probable clinical bioprosthetic aortic valve thrombosis, who reported good outcomes with warfarin at 39-month follow-up. The reversibility of bioprosthetic valve hemodynamic deterioration after anticoagulation strongly supports the probable diagnosis of clinical BPVT, in the absence of histopathology. Early moderate-to-severe hemodynamic valve deterioration warrants further investigations, including cardiac CT and sequential echocardiography, to investigate for probable subclinical BPVT and consideration of timely anticoagulation initiation to prevent thromboembolic events. Further studies are required to understand the natural history and valve-related consequences of subclinical BPVT, and the implications of anticoagulation.

## Supplementary Information

**Additional file 1.** Transoesophageal echocardiography prior to presentation. Transoesophageal echocardiography showed restrictions of the aortic leaflet opening, contributing to the raised transaortic gradient. There was no mass or vegetation visualized.

**Additional file 2.** Coronary angiography at presentation. Left anterior obliquecranial view of the coronary arteries. There is an embolus in the first diagonal branch with no evidence of underlying atherosclerosis of the left main and left anterior descending coronary arteries.

**Additional file 3.** Four-chamber view of transthoracic echocardiography at presentation. Transthoracic echocardiography showed reduced left ventricular systolic function.

## Data Availability

Some data are available upon request, while some data may not be available due to privacy and confidentiality of the patient.

## Abbreviations

AVA: Aortic valve area
CE: Coronary embolism
CT: Computed tomography
DPI: Dimensionless performance index
EF: Ejection fraction
MPG: Mean pressure gradient
NSTEMI: Non-ST-Elevation myocardial infarction
PPG: Peak pressure gradient
BPVT: Bioprosthetic valve thrombosis
TTE: Transthoracic echocardiography
TOE: Transoesophageal echocardiography
V_max_: Peak velocity
